# Pyruvate kinase M2 prevents apoptosis via modulating Bim stability and associates with poor outcome in hepatocellular carcinoma

**DOI:** 10.18632/oncotarget.3262

**Published:** 2015-03-02

**Authors:** Wen Hu, Shi-Xun Lu, Min Li, Chao Zhang, Li-Li Liu, Jia Fu, Jie-Tian Jin, Rong-Zhen Luo, Chris Zhiyi Zhang, Jing-Ping Yun

**Affiliations:** ^1^ Sun Yat-sen University Cancer Center, State Key Laboratory of Oncology in South China, Collaborative Innovation Center for Cancer Medicine, Guangzhou 510060, China; ^2^ Department of Pathology, Sun Yat-sen University Cancer Center, Guangzhou 510060, China

**Keywords:** PKM2, Bim, apoptosis, hepatocellular carcinoma

## Abstract

Pyruvate kinase M2 (PKM2) contributes to the Warburg effect, a hallmark of cancer. We showed that PKM2 levels were correlated with overall survival (hazard ration = 1.675, 95% confidence interval: 1.389–2.019, *P* < 0.001) and disease-free survival (hazard ration = 1.573, 95% confidence interval: 1.214–2.038, *P* < 0.001) in a cohort of 490 patients with HCC. The correlations were further validated in an independent cohort of 148 HCC patients. Multivariate analyses revealed that PKM2 was an independent indicator of poor outcome in HCC. The knockdown of PKM2 in HCC cells inhibited cell proliferation and induced apoptosis *in vitro* and *in vivo*. Bim siRNA markedly abolished the PKM2-depletion-induced apoptosis. PKM2 depletion decreased the degradation of Bim. In clinical samples, PKM2 expression was reversely correlated with Bim expression. Combination of PKM2 and Bim levels had the best prognostic significance. We suggest that PKM2 serves as a promising biomarker for poor prognosis of patients with HCC and its knockdown induces HCC apoptosis by stabilizing Bim.

## INTRODUCTION

Pyruvate kinase M2 (PKM2) is a rate-limiting enzyme in the process of transferring phosphoenolpyruvate (PEP) and ADP to pyruvate and ATP, respectively [1]. PKM2 contributes to the Warburg effect [2]. Previous studies demonstrated that PKM2 was mainly expressed in well-differentiated tissues [3]. Overexpression of PKM2 was detected in a various types of cancer, such as glioma [4] and tongue tumor [5]. Zhang *et al*. showed that high PKM2 expression was associated with shorter overall survival in esophageal squamous cell cancer [6]. Recently, up-regulation of PKM2 in hepatocellular carcinoma has been recently reported [7, 8].

PKM2 is capable of promoting the progression of human cancers. Yang *et al*. reported that nuclear PKM2 induced c-Myc expression by acting as a coactivator of β-catenin [4, 9]. Lee and colleagues showed that PKM2 enhanced Oct-4-mediated transcription by interaction with Oct-4 in glioma [10]. Recently, Jiang *et al*. demonstrated that PKM2 directly controlled cell-cycle progression by binding to Bub3 and phosphorylating it at Y207 [11]. On the other hand, Goldberg *et al*. reported that PKM2 silence by siRNA repressed cell growth by inducing apoptosis in cancer cells [12]. The molecular mechanism of PKM2-mediated apoptosis has not yet been investigated.

Apoptosis is a morphologically distinct form of programmed cell death, which is mainly governed by intrinsic and extrinsic apoptotic pathway [13]. The intrinsic pathway of apoptosis, controlled by mitochondrial outer membrane permeabilization (MOMP), has vital effect on tumor generation and progression [14]. It is well acknowledged that Bcl-2 family proteins are cable of modulating MOMP to determine the cell fate [15]. Bim, a member of Bcl-2 family proteins, possesses diverse pro-apoptotic activity [16]. The stability of Bim has been demonstrated to be regulated by protein kinase A (PKA) [17] and differentiation-related gene-1 (Drg1) [18]. Interacting with Bcl-xl and Mcl-1, Bim initiates cytochrome *c* release from mitochondria, activates Caspase 9 and consequently triggers mitochondrial apoptotic pathway [19].

In this study, the expression of PKM2 and its clinical significance in HCC were determined. The role of PKM2 in HCC cell apoptosis and its relationship with Bim were further investigated. Our data suggest PKM2 as a promising biomarker for prognosis of patients with HCC.

## RESULTS

### PKM2 is overexpressed in HCC cell lines and tissues

**Figure 1:**
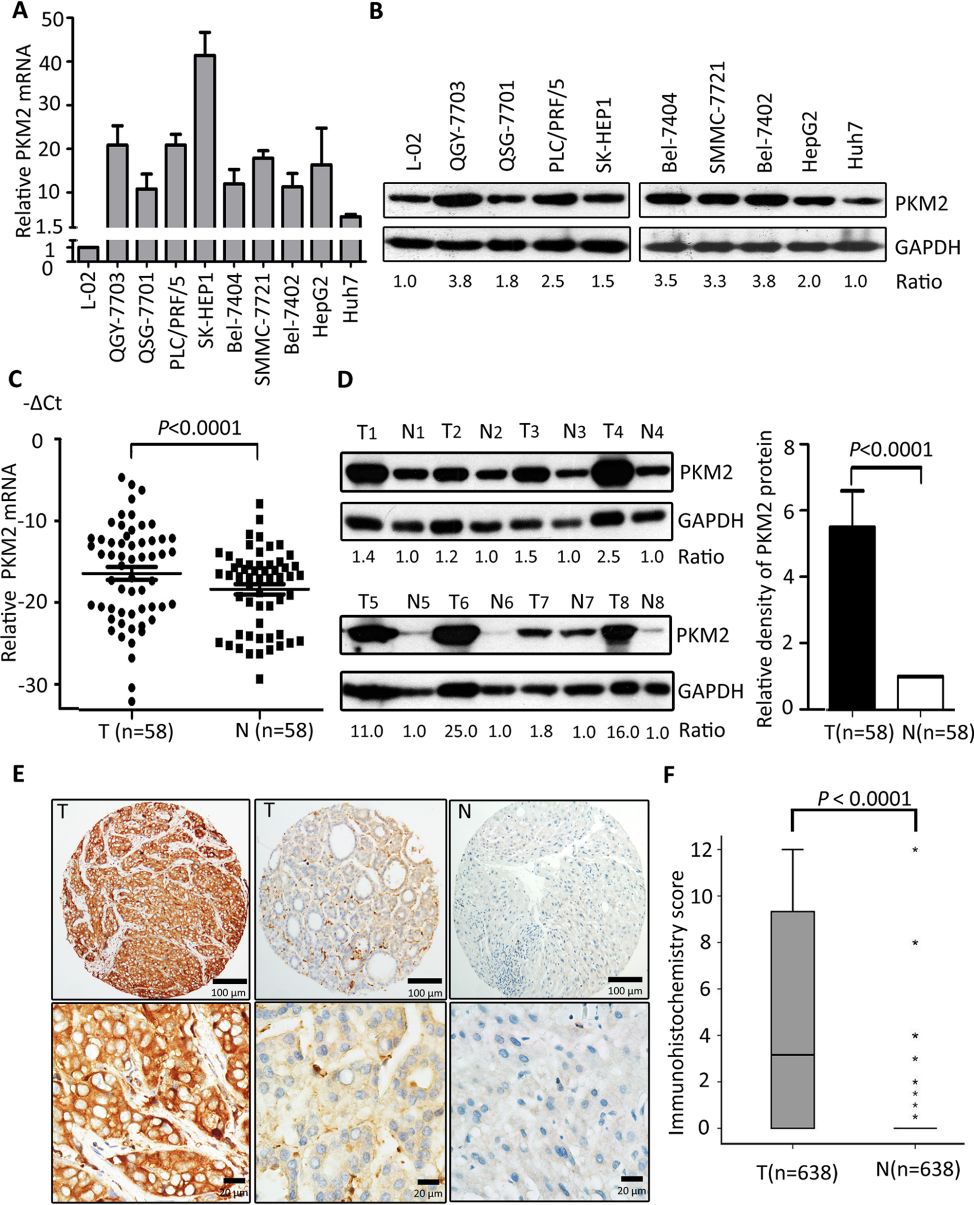
PKM2 is overexpressed in HCC cell lines and tissues **(A)** Expression of PKM2 mRNA was detected in 9 HCC cell lines by qRT-PCR. Immortalized liver cell line L-02 was used as control. **(B)** The relevant expression of PKM2 in HCC cell lines was examined by western blot. **(C)** PKM2 mRNA level was determined in 58 pairs of fresh primary HCC tissues (*P* < 0.0001, Wilcoxon matched-paired test) (T, tumorous tissue; N, nontumorous tissue). **(D)** The expression level of PKM2 protein in 58-paired samples was also examined by western blot. Representative results and the ratio of T/N were shown. Increased expression of PKM2 protein in tumorous tissues was indicated by histogram (*P* < 0.0001, Wilcoxon matched-paired test). **(E)** PKM2 expression in 638 HCC tissues was determined by IHC. Representative images of strong/weak staining in HCC tissue and negative staining in the nontumorous tissue were shown. **(F)** The box plot showed the IHC score of PKM2 in 638 HCC cases. Data are mean ± SEM (**P < 0.0001, Wilcoxon matched-paired test).

The expression of PKM2 in HCC cells was firstly determined. Results showed that PKM2 expression at both mRNA and protein levels in 9 HCC cells was noticeably up-regulated, compared to the immortalized hepatic cell L-02 (Figure 1A&1B). In HCC fresh samples, PKM2 mRNA was significantly overexpressed in tumorous tissues (Figure 1C). Consistently, the protein level of PKM2 was markedly increased in 54 out of 58 (93.1%) primary HCC cases, compared to the corresponding nontumorous tissues (Figure 1D and Supplementary Figure 1). In a large cohort of 638 HCC patients, results of immunostaining showed that PKM2 expression in HCC tissues was remarkably higher than that in the adjacent normal liver tissues (Figure 1E&1F, *P* < 0.0001, Wilcoxon matched-paired test).

### High PKM2 expression is closely correlated with worse clinical outcomes

We next investigated the relationship between PKM2 expression and clinicopathologic variables. In the training cohort of 490 patients, high PKM2 expression was more likely to present advanced clinical characters, including higher serum α-Fetoprotein level (*P* < 0.001), advanced clinical stage (*P* = 0.004), vascular invasion (*P* = 0.001) and tumor size (*P* = 0.042). This was further validated in another independent cohort of 148 patients with HCC (Supplementary Table 2).

**Figure 2:**
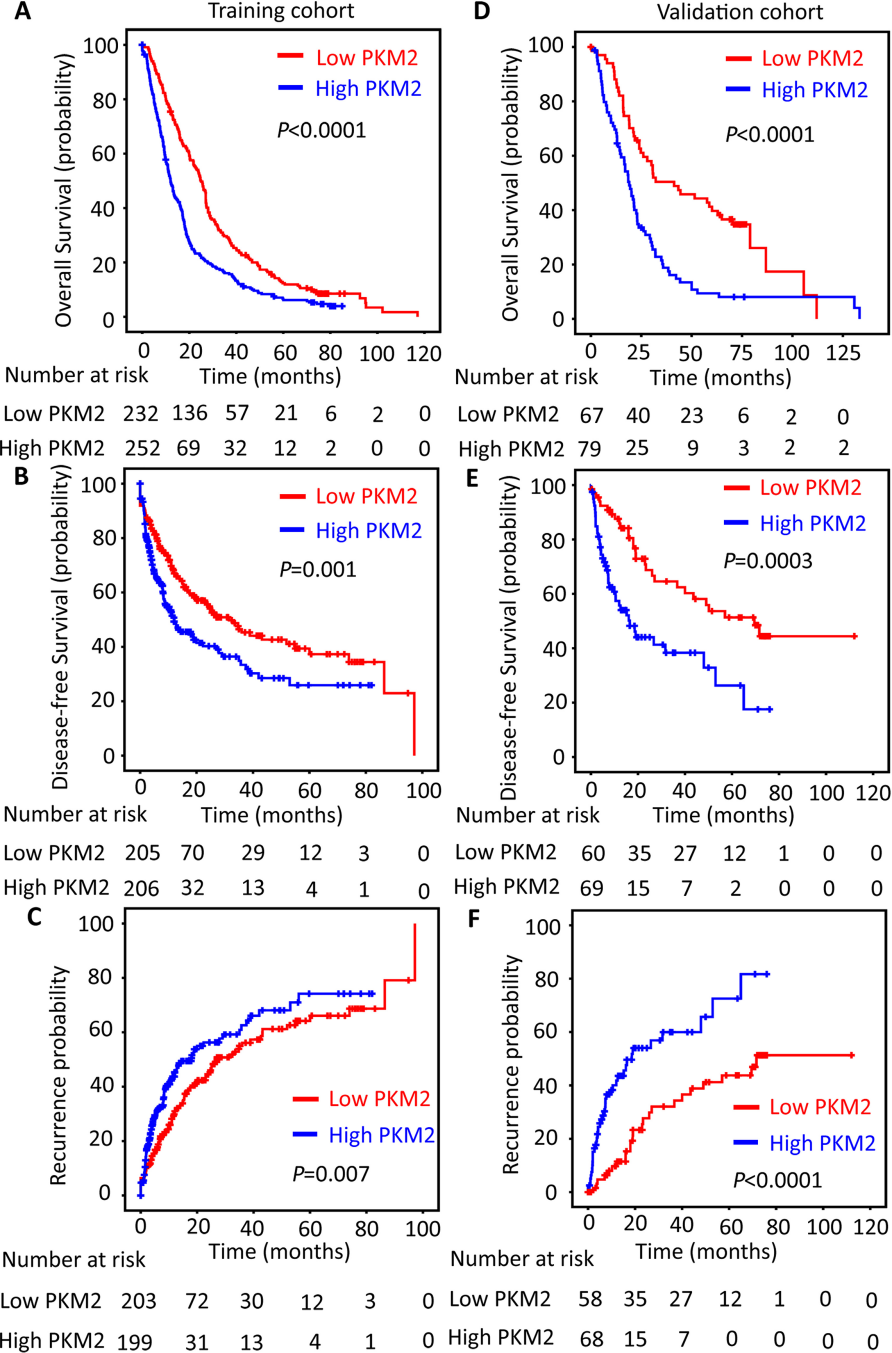
PKM2 expression is reversely correlated with outcomes of HCC patients The HCC patients in the training (*n* = 490) and validation (*n* = 148) cohort were stratified according to the expression of PKM2. Kaplan–Meier analysis disclosed the relationship of PKM2 expression and the overall survival **(A&D)**, disease-free survival **(B&E)** and recurrence probability **(C&F)** of HCC patients (log-rank test).

The prognostic significance of PKM2 was also determined. Kaplan–Meier analysis indicated a significantly better prognosis in HCC cases with low PKM2 expression, in terms of overall survival (*P* < 0.0001), disease-free survival (*P* = 0.001) and recurrence-free survival (*P* = 0.007) in the training cohort. On the contrary, high PKM2 expression tended towards unfavorable prognosis (log-rank test, Figure 2A–2C). Consistently, increase of PKM2 was associated with inferior overall survival (*P* < 0.0001), disease-free survival (*P* = 0.0003) and recurrence-free survival (*P* < 0.0001) in the validation cohort (log-rank test; Figure 2D–2F). Multiple Cox regression analysis showed that PKM2 retained an independent factor for both overall survival and disease-free survival in both training and validation cohorts (Supplementary Table 3&4).

Consistent with the results in the individual cohort, high PKM2 expression favored a substantially shorter overall survival (*P* < 0.0001), disease-free survival (*P* < 0.0001) and recurrence-free survival (*P* < 0.0001) in the overall cohort of 638 patients with HCC (Supplementary Figure 2; log-rank test). High PKM2 expression was also closely associated with higher serum α-Fetoprotein level (*P* < 0.001), advanced clinical stage (*P* < 0.0001), larger tumor (*P* = 0.005), vascular invasion (*P* < 0.0001) and more multiple nodular tumor (*P* = 0.027) in all cases enrolled into this study (Supplementary Table 5). Importantly, PKM2 was shown to be an independent prognostic factor for overall survival (hazard ratio = 1.522; 95%CI: 1.282–1.807; *P* < 0.0001) and disease-free survival (hazard ratio = 1.639; 95%CI: 1.298–2.070; *P* < 0.0001) in multivariate analysis (Supplementary Table 6).

### PKM2 knockdown inhibits HCC cell proliferation and induces apoptosis

**Figure 3:**
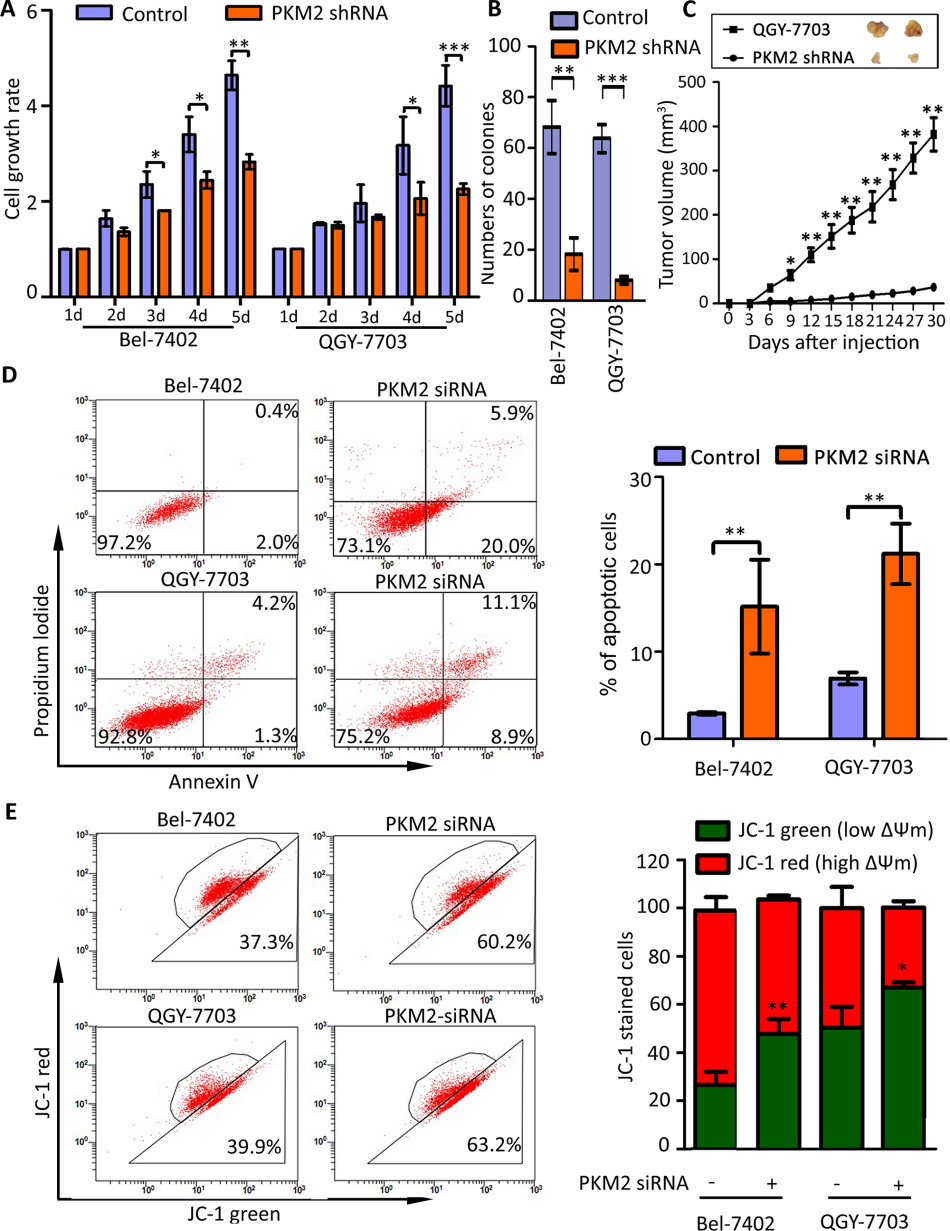
PKM2 knockdown inhibits cell proliferation and induces apoptosis **(A)** PKM2 knockdown impaired cell viability. Cells stably transfected with PKM2 shRNA or scrambled shRNA were cultured in 96-well plate for 5 d. Cell viability was measured by MTT Assays. **(B)** PKM2 depletion weakened monoclonal formation ability of HCC cells. Number of colonies was shown. **(C)** PKM2 knockdown inhibited tumor growth *in vivo*. Stable cell lines were subcutaneous injected into the right flank of null mice. Tumors were sectioned at day 28. The tumor volumes were recorded every three days. **(D)** PKM2 silence induced apoptosis in HCC cells. Cells were transfected with negative or PKM2 siRNA for 48 hours, and then stained with Annexin V and Propidium Iodide (PI) (left panel). Percentage of apoptotic cells were recorded (right panel). **(E)** PKM2 siRNA induced the decrease of mitochondrial membrane potential. Cells were transfected with PKM2 siRNA for 48 h and stained with JC-1 for 15 min. The mitochondrial transmembrane potential (ßψm) was determined by FACS (left panel). Graph depicts mean ± SEM of three independent experiments (right panel). All *P < 0.05, **P < 0.01, ***P < 0.001.

Furthermore, we determined the effect of PKM2 knockdown on HCC cell growth. Results showed that PKM2 depletion significantly inhibited HCC cell proliferation and impaired the colony formation ability *in vitro* (Figure 3A&3B). *In vivo* data revealed that cells with PKM2 knockdown exhibited an obvious decrease in tumor formation capacity, compared with control group (Figure 3C). Further study showed that PKM2 silenced induced HCC cell apoptosis (Figure 3D). The average percentages of apoptotic cells were 15.8% and 21.2%, respectively in Bel-7402 and QGY-7703 cells, while the numbers of control cells were 2.9% and 6.9% (Figure 3D). Result of JC-1 staining presented that PKM2 knockdown increased the red-to-green fluorescence intensity ratio, indicating damage of mitochondrial transmembrane potential which is one of the signature of cell apoptosis (Figure 3E).

### Bim is essential for PKM2-depletion-induced apoptosis

**Figure 4:**
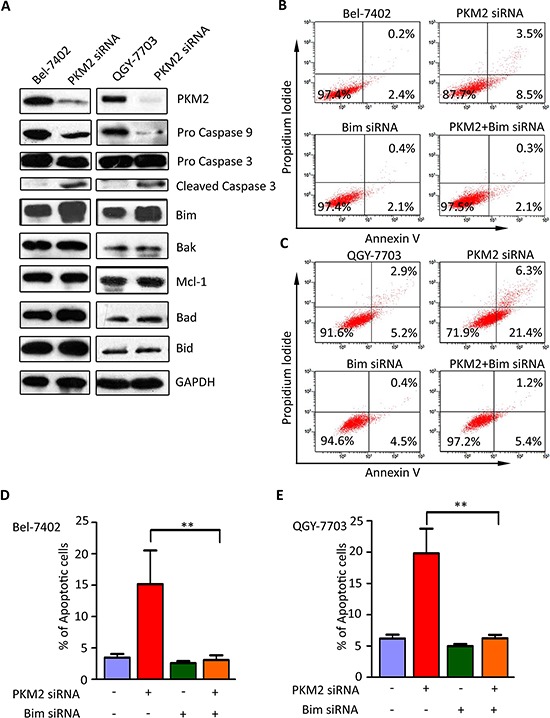
Bim is essential for PKM2–depletion-induced apoptosis **(A)** Silence of PKM2 led to increase of Bim expression and activation of Caspase 9. HCC cells were transfected with PKM2 or negative control siRNA for 48 h. Expressions of Caspase 3, Caspase 9 as well as Bcl-2 family proteins, including Bid, Bim, Mcl-1, Bad and Bak, were examined by western blot. GAPDH was used as loading control. **(B–E)** Bim siRNA attenuated apoptosis caused by PKM2 siRNA. PKM2 and Bim siRNA were co-transfected into QGY-7703 and Bel-7402 cells for 48 h. Apoptosis was determined by flow cytometry. All **P < 0.01, paired student *t*-test.

To assess examined whether Caspase pathway was involved in PKM2-depletion-induced apoptosis, we conducted the following experiments. Following PKM2 siRNA treatment for 48 h, both Caspase 9 and Caspase 3 were activated. Cleaved form of Caspase 3 was found in the cells transfected with PKM2 siRNA (Figure 4A). Since Bcl-2 family proteins play important roles in mitochondrial-mediated and caspase-mediated apoptosis, expression profile of Bcl-2 family members was determined. For further study about the specific apoptotic pathway which is triggered by PKM2 deficiency, we determined the expression alteration of the Bcl-2 family in PKM2-knock out hepatoma cells. In this study, the pro-apoptotic protein Bim was notably upregulated in group cells when PKM2 knocked down. Nevertheless, expressions of other apoptosis-related proteins including Bak, Mcl-1, Bad and Bid, remained unchanged (Figure 4A). Bim siRNA markedly abolished the apoptosis driven by PKM2 depletion (Figure 4B–4E), which suggested the critical role of Bim in PKM2-mediated apoptosis.

### PKM2 knockdown attenuates the degradation of Bim

**Figure 5:**
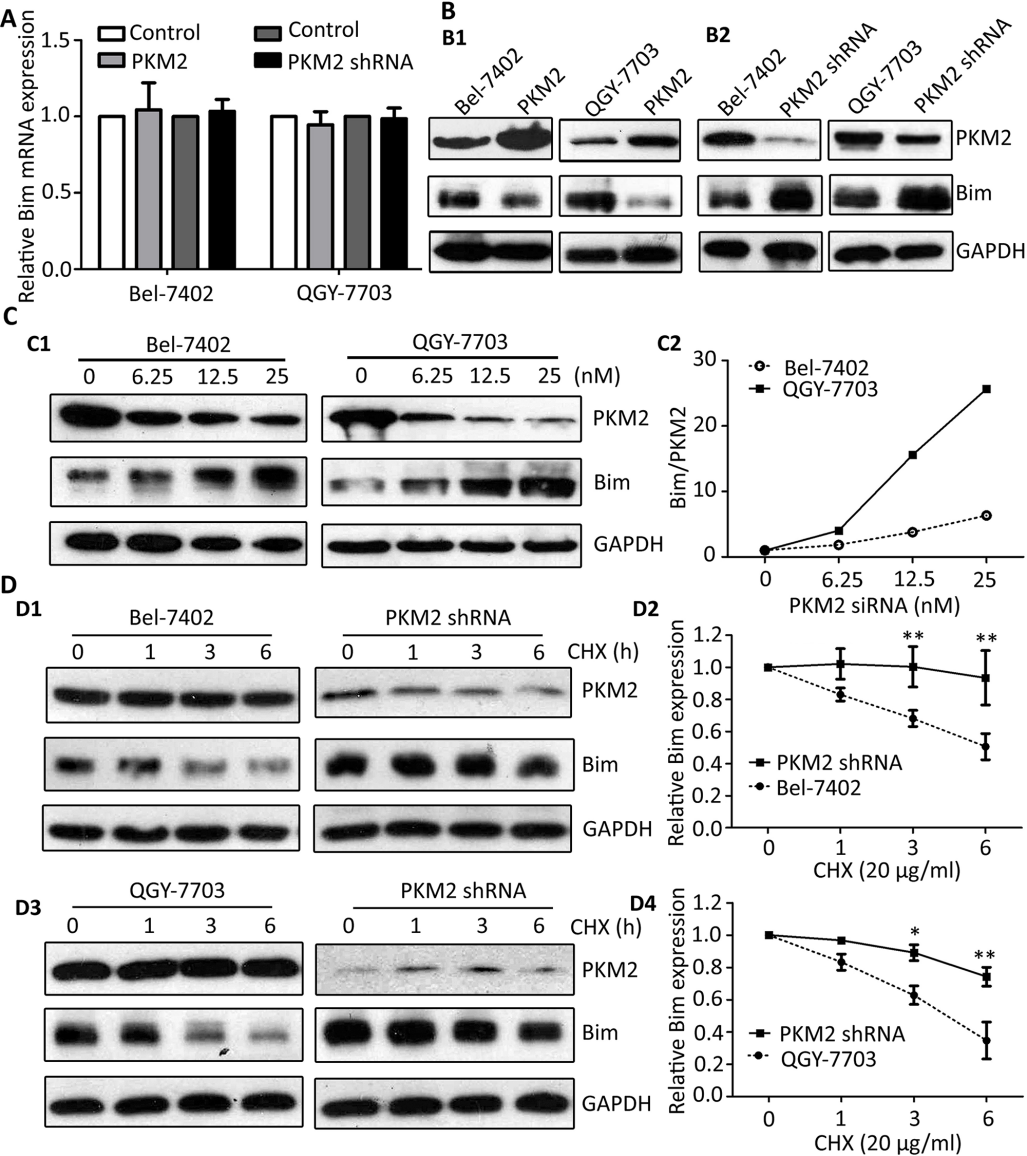
PKM2 knockdown attenuates the degradation of Bim **(A)** PKM2 did not affect the mRNA level of Bim. Bim mRNA expression in cells with PKM2 overexpression or knockdown was determined by qRT-PCR. **(B)** PKM2 regulated the expression of Bim protein. Level of Bim protein was examined in cells transfected with PKM2 (B1) and PKM2 shRNA (B2). **(C)** PKM2 silence increased Bim expression in a dose-dependent manner. Cells were treated with various concentration of PKM2 siRNA for 48 h, and the expressions of PKM2 and Bim were determined (left panel). The relative Bim/PKM2 ratio was calculated (right panel). **(D)** PKM2 contributed to the stability of Bim protein. Stable cells with PKM2 knockdown were treated with 20 ug/ml CHX for indicated times. The expression of Bim was detected (D1 and D3). The decrease of Bim protein was normalized and shown (D2 and D4). GAPDH was used as loading control. At least three experiments were performed. Data are mean ± SEM (*P < 0.05, **P < 0.01).

We have found that knockdown PKM2 can alter the expression of Bim (Figure 4A), so we tested the relationship of Bim expression and PKM2. As the results showed, no significant change of Bim mRNA level in both PKM2-elevated and PKM2-depletion HCC cells was noticed (Figure 5A). However, Bim protein expression was substantially restrained in PKM2-overexpressed cells (Figure 5B1) whereas it was increased in PKM2-depletion hepatoma HCC cells (Figure 5B2). Moreover, Bim was promoted up-regulated by PKM2-specific siRNA in a dose-dependent manner (Figure 5C1). The Bim/PKM2 protein ratio was gradually elevated upon the increasing dose of PKM2 siRNA (Figure 5C2).

Based on our findings, we assumed that PKM2 might regulate Bim expression at post-transcriptional level. Therefore, we next examined whether PKM2 affected the half-life of Bim by using Cycloheximide (CHX, a protein synthesis inhibitor). Data showed that Bim was fast decreased with 20 μg/ml CHX treatment for 6 h in the control cells. Once PKM2 was knocked down, Bim degradation was dramatically attenuated in both cell lines (Figure 5D1–5D2) and QGY-7703 cells (Figure 5D3–5D4).

### PKM2 expression is reversely correlated with Bim expression in clinical samples

**Figure 6:**
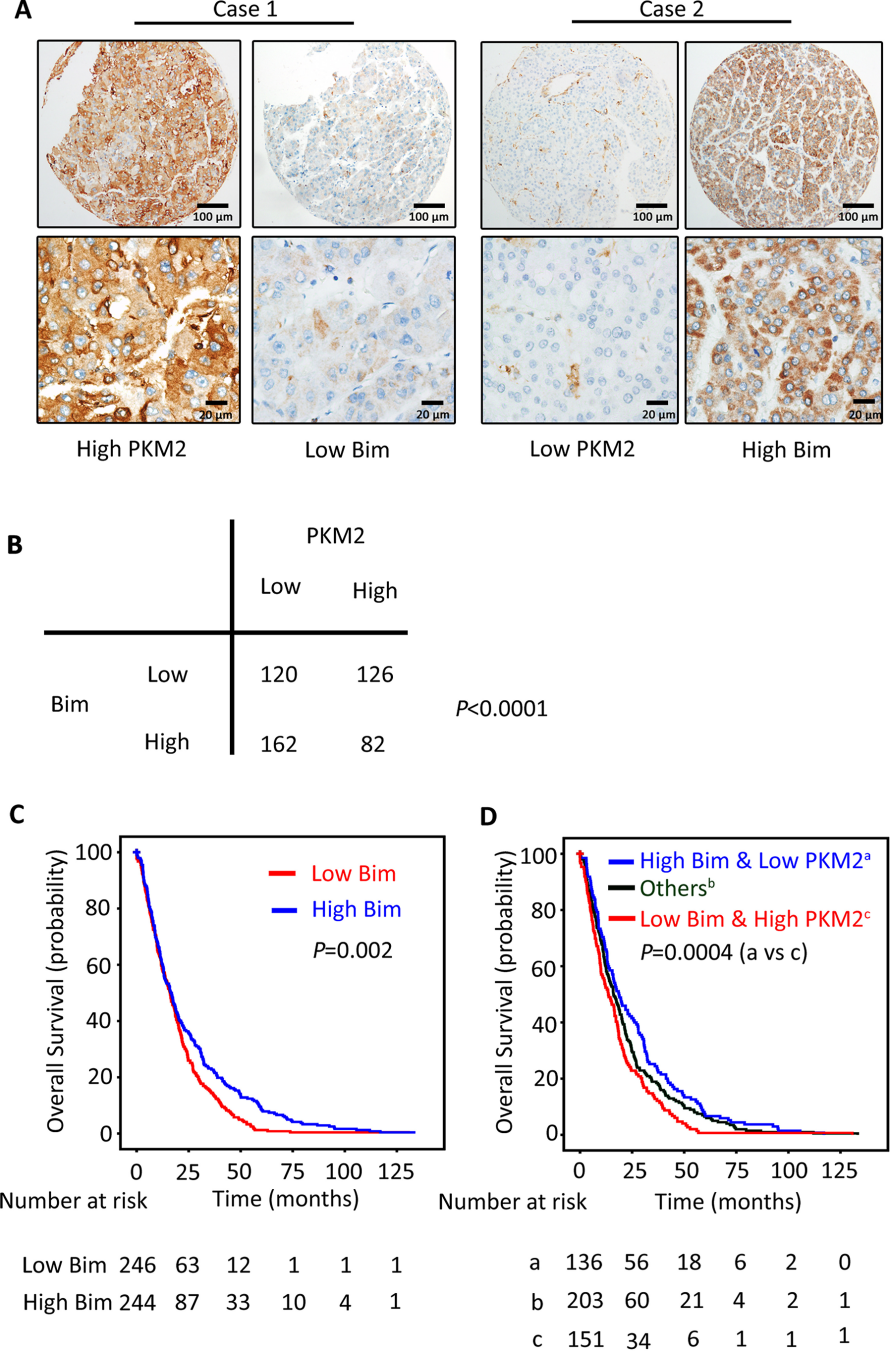
PKM2 expression is reversely correlated with Bim expression in clinical samples **(A)** The relationship of PKM2 and Bim in 490 patients with HCC was determined by IHC. Representative immunohistochemical images of PKM2 and Bim were shown. **(B)** Spearman correlation analysis showed the relationship of PKM2 and Bim expression. The *p* value was generated using χ^2^ test. **(C)** The prognostic value of Bim was determined by Kaplan–Meier analysis. **(D)** HCC cases were divided into 3 groups according to the combination of PKM2 and Bim expression. Kaplan–Meier analysis was conducted to estimate of overall survival of the patients in the 3 groups.

To explore the relationship between PKM2 and Bim expression in clinical samples, the expression of PKM2 and Bim was analyzed in a large cohort of 490 patients with HCC. Results showed cases with high PKM2 expression in tumor tissues were frequently with low Bim expression (Figure 6A). Statistically, PKM2 expression was significantly reversely associated with Bim expression (Figure 6B, *P* < 0.0001).

Although it was not significantly connected with clinical parameters in HCC (Supplementary Table 7), Bim expression was of prognostic value. Kaplan–Meier survival analysis displayed that high level of Bim in HCC cases was associated with longer overall survival (Figure 6C, *P* = 0.002), but not with disease-free survival (Supplementary Figure 3A, *P* = 0.981) and recurrence-free survival (Supplementary Figure 3B; *P* = 0.399). Multivariate analysis indicated Bim was also an independent predictor for overall survival in HCC (Supplementary Table 8). Interestingly, combination of expressions of PKM2 and Bim was of better prognostic value. Patients with high PKM2 and low Bim expression survived shortest (Figure 6D).

## DISCUSSION

PKM2 has been demonstrated to be notably up-regulated in various types of cancers and responsible for their poor prognosis, such as gastric cancer [20] and prostate cancer [21]. In this study, we found that in a large cohort of 638 patients with HCC, PKM2 expression was markedly increased and closely correlated with malignance clinical characteristics, including higher serum α-Fetoprotein level, advanced clinical stage, vascular invasion as well as tumor size. Furthermore, PKM2 served as an independent risk factor indicating unfavorable prognosis. Our data suggest PKM2 may be clinically significant, not merely for the reason that large samples were enrolled in our research but also for the reason that HCC lacks a common biomarker of poor prognosis. Similarly, newly-published data showed that PKM2 was overexpressed in a cohort of 721 patients with HCC and indicated unfavorable prognosis in HCC [7]. It is noteworthy that patients in our study were from South China, whereas the cohort in Liu's study was from North China. As a result, these two original studies can be considered as two independent studies with large cohort of patients with HCC from two independent centers in mainland China. On the other hand, we showed that high PKM2 expression was positively related to unfavorable disease-free survival. This may indicate that PKM2 could be useful to identify patients who should be closely under surveillance for tumor recurrence.

It has been reported that HCC proliferation is mainly due to glucose metabolism in which PKM2 plays an important role [22]. The activity of PKM2 in cell proliferation is attributed to its nuclear localization. Gao et al. showed that nuclear PKM2 phosphorylated stat3 to up-regulate MEK5 to promote cell proliferation [23]. Yang and colleagues demonstrated that PKM2 phosphorylated histone H3 at T11 to modulate the expression of cyclin D1 and c-myc [24]. In another study, PKM2 was shown to regulate Bub3 to affect the cell cycle in tumor cells [25]. Wang *et al*. found that upregulation of PKM2 by Spry2 accelerates AKT-induced hepatocarcinogenesis [26]. In our study, PKM2 expression was positively correlated to the expression of Ki-67 in 103 clinical samples (data not shown), indicating the role of PKM2 in modulating HCC cell proliferation. Since PKM2 was predominantly expressed in cytoplasm in our study, we focused on the role of PKM2 in cell apoptosis. Steták *et al*. reported that the PKM2 markedly induced programmed cell death in Cos-7 cells [27]. In the present study, we demonstrated that PKM2 silence induced decrease of mitochondrial membrane potential, activation of Caspase 3 and cell apoptosis. Our data may complement the powerful function of PKM2 in regulating cell death in HCC.

Our data showed that PKM2 knockdown induced apoptosis by upregulation of Bim. Interestingly, Shimada *et al*. reported that PKM2 inhibited apoptosis of intestinal epithelial cells by upregulating anti-apoptotic protein Bcl-xl [28]. In a following study, Kwon *et al*. proved that PKM2 promoted cell growth in gastric cancer by inducing Bcl-xl expression at transcriptional level [29]. The transcriptional activity of PKM2 was also reported in colon cancer, showing that PKM2 interacted with TGIF2 to transcriptionally regulated TGF-β signaling [30]. In our study, PKM2 knockdown resulted in no significant change of Bim mRNA. Instead, the stability of Bim was highly maintained, whereas direct binding of PKM2 and Bim was not observed (data not shown), which suggested PKM2 may indirectly regulate Bim expression in a post-transcriptional manner. It has been well studied that Bcl-xl interacted with Bim to suppress cell apoptosis [31, 32]. Although no evidence showed that Bcl-xl could affect the expression of Bim, whether Bcl-xl was involved in the regulation of Bim stability by PKM2 requires future study.

The reverse connection of Bim and PKM2 was further confirmed in clinical samples. HCC patients with low PKM2 were frequently accompanied with high Bim expression. The prognostic value of Bim was also determined. Patients with high Bim expression survived longer in our study cohort. Furthermore, combination of PKM2 and Bim expression was of better prognostic significance for HCC patients. Based on that PKM2 acts as an oncogene and Bim plays a role of tumor suppressor in HCC, it is not surprising to find that patients with high PKM2 and low Bim expression should be more clinically followed up. Hence, our study provides a promising strategy for clinical management for the postoperative HCC patients.

In summary, our data show that expression of PKM2 expression is remarkably increased and robustly bounded up with clinicopathologic parameters and unfavorable clinical outcome of patients with HCC. PKM2 depletion resulted in cell apoptosis by stabilizing proapoptotic protein Bim. This study suggested PKM2 as a promising biomarker for poor prognosis of HCC.

## MATERIALS AND METHODS

### Chemicals and reagents

Dulbecco's modified Eagle's medium (DMEM) and RPMI-1640 were products of Gibco (Gibco, Gaithersburg, MD, USA). The RIPA buffer supplemented with a protease inhibitor cocktail (P8340) were products of Sigma-Aldrich (MA, USA). The phosphatase inhibitors were purchased from Roche Diagnostics (Shanghai, China). Monoclonal antibodies against PKM2, Bim, Caspase 3, Caspase 9, Bad, Bcl-2, Mcl-1, Bid, Bak were purchased from Cell Signaling Technology (USA). Monoclonal antibodies against GAPDH and HRP-conjugated anti-mouse and anti-rabbit antibodies were purchased from Santa Cruz Biotechnology Inc from Genetime Co. (Calif., USA). Annexin V-FLOUS double Staining kit was purchased from Roche (Basel., Switzerland). Other routine laboratory reagents were obtained from commercial sources of analytical (Guangzhou, China).

### Cell lines and cell culture

Human hepatic carcinoma cell lines SK-Hep1, PLC/PRF/5, Huh7 were acquired from American Type Culture Collection (ATCC, Manassas, VA), which was cultured with Dulbecco's modified Eagle's medium (DMEM) (Gibco, Gaithersburg, MD, USA) supplemented with 10% heat-inactivated fetal bovine serum (FBS, Hyclone, Logan, UT). Human hepatic carcinoma cells QGY-7703, SMMC7721, Bel-7404 and Bel-7402 were purchased from the Cell Resource Center, Chinese Academy of Science Committee (Shanghai, China). Human normal liver cell L-02 was also obtained from the Cell Resource Center, Chinese Academy of Science Committee (Shanghai, China). These cells were cultured in RPMI-1640 medium containing 10% FBS. All cells were cultivated at 37°C in a humidified atmosphere of 5% CO_2_.

### Western blot analysis

The whole cell extracts were collected in cell lysis buffer (1 × PBS, 1% Nonidet P-40, 0.5% sodium deoxycholate, 0.1% SDS, 100 mg/mL phenylmethylsulfonyl fluoride, 10 mg/mL aprotinin, 10 mg/ml leupeptin). Equal amounts of protein (30 μg) were resolved by SDS-PAGE and then electrophoretically transferred onto PVDF membranes. After blocked in 5% non-fat milk 1 h at room temperature, the membranes were incubated with appropriately diluted primary antibodies overnight at 4°C. After washed thrice with TBST, the membranes were incubated with HRP-conjugated secondary antibody at 1:20000 dilutions for 1 h at room temperature. The membranes were visualized by the enhanced Phototope TM-HRP Detection Kit (Cell Signaling, USA) and exposed to Kodak medical X-ray processor (Carestream Health, USA). GAPDH was used as the loading control.

### Patients and tissue specimens

A total of 638 surgical resected specimens of primary liver cancer between January 2000 and December 2010 were collected from the archives of the Department of Pathology of the Sun Yat-sen University (Guangzhou, China). The patients included 578 (90.6%) female and 60 (9.4%) male and the median age is 49 years (age range from 13 to 68 years). The mean follow-up time is 25.9 months. None of the patients received any chemotherapy or radiotherapy before operation. All diagnosis were made by expert pathologists from Sun Yat-sen University (Guangzhou, China), according to the criteria for terminology established by the International Working Party. We randomly divided these cases into a training cohort (*n* = 490, 76.8%) and an independent validation cohort (*n* = 148, 23.2%). No statistically significant differences were detected in most relevant clinical data between training cohort and validation cohort, including tumor size, hepatitis history, age, gender, serum AFP level, tumor differentiated degree, clinical stage and incidence of cirrhosis and vascular invasion (Supplementary Table 1, all *P* > 0.05) although a little more patients with single tumor nodule were divided into the validation cohort. This study has been approved by the Institute Research Medical Ethics Committee of SYSUCC.

### Tissue microarray (TMA) and immunohistochemistry

The TMA slides included 638 HCC and adjacent nontumorous liver tissues. Using a tissue array instrument (Minicore excilone, Minicore, British), each tissue core with a diameter of 0.6 mm was punched from the marked areas and re-embedded. All specimens were fixed at 4% paraformaldehyde in 0.1 M phosphate buffer for 24 h and embedded in paraffin wax. Then the paraffin-embedded HCC sections were sliced into 4 μm and mounted onto glass slides. After dewaxed, the slides were treated by 3% hydrogen peroxide in methanol and blocked by a biotin-blocking kit (DAKO, Germany). After blocking, the slides were incubated with PKM2 antibody (1:1000, Cell Signaling Technology, USA) or Bim antibody (1:200, Cell Signaling Technology, USA) overnight in a moist chamber at 4°C. After washed in PBS for three times, the slides were incubated with biotinylated goat anti-rabbit/mouse antibodies for 1 h. Then the slides were stained with the DAKO Liquid 3,’3-diaminobenzidine tetrahydrochloride (DAB). Finally, the slides were counter stained with Mayer's hematoxylin and observed in Olmpus Scaper.

The protein level was determined by Semi-quantitative immunohistochemistry (IHC) detection. The positively-stained was scored as follow: “0” (less than 5% positively-stained cells), “1” (6–24% of positively-stained cells), “2” (25–49% of positively-stained cells), “3” (50–74% of positively-stained cells) and “4” (75%–100% of positively-stained cells). Intensity was scored was according to the standard: “0” (negative staining); “1” (weak staining); “2” (moderate staining) and “3” (strong staining). The final score was served by multiplying the percentage score by the staining intensity score. The scores were independently decided by two pathologists (Dr Jing-Ping Yun and Dr Rong-Zhen Luo). The median IHC score was chosen as the cut-off value for defining high and low expression. In this study, the cut-off value for PKM2 and Bim was 3 and 4.5.

### Quantitative real-time PCR (qRT-PCR)

Total RNA was extracted from clinical samples and cultured cells using Trizol reagent (BIOO Scientific Co., USA) following manufacture instruction. The reverse transcription with random primers was done by M-MLV Reverse Transcriptase (Promega Inc., USA) according to the manufacturer's instructions. SYBR green-based real-time PCR as carried out to measure levels of the corresponding *PKM2*, *Bim* and *β-actin* by the Strata gene Mx3000P Real-time PCR system. Primers were designed as follows: *PKM2*, forward :5′-CCATTACCAGCGACCCCACAG-3′ and reverse: 5′-GGGCACGTGGGCGGTATCT-3′; *Bim*, forward: 5′-TAAGTTCTGAGTGTGACCGAGA-3′ and reverse: 5′-GCTCTGTCTGTAGGGAGGTAGG-3′; *β-actin*, forward: 5′-CACCATGAAGATCAAGATCATTGC-3′ and reverse: 5′-GGCCGGACTCATCGTACTCCTGC-3′. The qRT-PCR reactions were done 95°C for 10 min for initial denaturation, and then 95°C for 30 seconds, 60°C for 30 seconds, 72°C for 30 seconds and a final extension of 10 min for 40 cycles. SDS 2.3 software (Applied Biosystems) was used to quantify and analyze the relative mRNA levels. Relative quantification of PKM2 and Bim mRNA was performed using the 2^−ΔΔCt^ method. The experiments were done at least thrice independently and all samples were in triplicate.

### Plasmid construction and transfection

The pGIPZ vector, pGIPZ PKM2 shRNA, pcDNA 3.1/hygro(+) vector and pcDNA 3.1/hygro(+)-PKM2 K367M were kindly provided by professor Lu [24]. All of the recombinant plasmids were confirmed by sequencing. We constructed the plasmids into QGY-7703 and Bel-7402 cells by lentiviral transduction. For stably transfection, 4 μg of plasmids pGIPZ vector and pGIPZ PKM2 shRNA were transfected into QGY-7703 and Bel-7402 cells by mixing with Lipofectamine™ 2000 (Invitrogen; Carlsbad, CA, USA) reagent according to the instructions of manufacturer. 48 hours later, we added 1 mg/ml of puromycin (Sigma Aldrih, MA, USA) into well for selection for 2 weeks. Finally, the puromycin-resistant cell population was tested for the expression of PKM2 by western blot as previously described. For transient transfection, various concentrations of PKM2 siRNA and scrambled siRNA (6.25, 12.5 and 25 nM) were transfected into QGY-7703 and Bel-7402 cells using Lipofectamine™ RNAiMAX (Invitrogen, Carlsbad, CA, USA). The whole cell extracts were collected after 24 or 48 h for immunoblotting. The siRNAs used in this study are listed below. PKM2 siRNA: 5′-CCAUAAUCGUCCUCACCAATT-3′; Bim siRNA: 5′-GACCGAGAAGGUAGACAAUUU-3′; Negative control: 5′-UUCUCCGAACGUGUCACGUTT-3′.

### Apoptosis assays

To detect the apoptosis induced by PKM2, we first transfected QGY-7703 and Bel-7402 cells with PKM2 siRNA and scrambled siRNA for 48 h. Then, the cells were collected by centrifugation and treated with Annexin V-FITC and propidium iodide (Annexin V-FLUOS Staining kit from Roche) for 15 min at room temperature in the dark according to the manufacturer's protocol. The cell suspension was immediately analyzed by flow cytometry (Beckman-Coulter, Inc, Fullerton, CA, USA) to analyze cell apoptosis. At least 10 000 events were collected for each sample at a flow rate of 100 cells per second.

For mitochondrial staining, each sample was incubated with JC-1 (Sigma–Aldrich, MO) at 37°C in the dark for 15 min and then washed twice with PBS. The samples were immediately analyzed using FCM (Beckman-Coulter, Inc, Fullerton, CA, USA). At least 7000 events were collected for each sample at a flow rate of 100 cells per second. The interest cell population was gated on the forward and side scatter to exclude debris and aggregates. For JC-1 fluorescence analysis, the FL1 and FL2 channels were used for detecting the green fluorescence (480–530 nm) and orange-red fluorescence (580–630 nm) [33, 34]. The mitochondrial trans-membrane potential (ßψm) was characterized by the ratio of red/green fluorescence.

### Cell viability assay

Briefly, the cells were collected and seeded in 96-well plates at a density of 3.0 × 10^3^ per well. At specific time according to the results, MTT (0.5 mg/mL, 100 μL) was added into each well and 4 h later, the medium was discarded and 150 μL DMSO was added into the wells. Finally, optical density was measured at 540 nm with background subtraction at 670 nm by Model 550 Microplate Reader (Bio-Rad, Hercules, CA, USA). Experiments were performed at least three times.

### Colony formation assay

The long-term survival of cells with stable transfection was detected by colony formation assay. Briefly, the cells were counted and plated into 6-well plates at a density of 1000 cells per well. After 10–14 days of incubation in complete culture medium, the cells colonies were fixed and stained with crystal violet solution (0.1% crystal violet, 10% formaldehyde) for 30 min at room temperature. After washed with PBS, the plates were air dried and scanned. The number of colonies were calculated by Image J software (NIH, Bethesda, MD, USA).

### Animals and tumor xenograft experiments

Athymic nude mice (5 to 6 weeks, weighing 18–22 g) were bred at the animal facility of the Center of Experimental Animals, Sun Yat-Sen University (China). Briefly, each nude mice was implanted subcutaneously (sc) under the right armpits with 4 × 10^6^ QGY-7703 cells containing a Amp-inducible scrambled or PKM2 knockdown sequence (pGIPZ PKM2 shRNA). Tumor size and body weight were measured every 2 days with digital calipers. Volumes were calculated using the following formula: volume = length × width^2^ × 0.5. Four weeks later, the mice were sacrificed and tumors were collected for further measurement. All animal studies were conducted with the approval of the Medical Experimental Animal Care Commission of Sun Yat-sen University Cancer Center.

### Statistical analysis

All results were depicted as mean values ± standard deviations (SDs). The statistical software SPSS16.0 was used in data processing and analyzing. Statistical analysis of the differences among various treatment and control groups was done using Student's *t*-test. *P* < 0.05 was considered as significant.

## SUPPLEMENTARY FIGURES AND TABLES


