# Supplementary material for: Pyruvate kinase M2 prevents apoptosis via modulating Bim stability and associates with poor outcome in hepatocellular carcinoma
Source: Oncotarget. 2015 Mar 2;6(9):6570–83. doi: 10.18632/oncotarget.3262 (PMC4466635; doi:10.18632/oncotarget.3262)
Supplement: Supplementary file 1 [file oncotarget-06-6570-s001.pdf]

SUPPLEMENTARY FIGURES AND TABLES

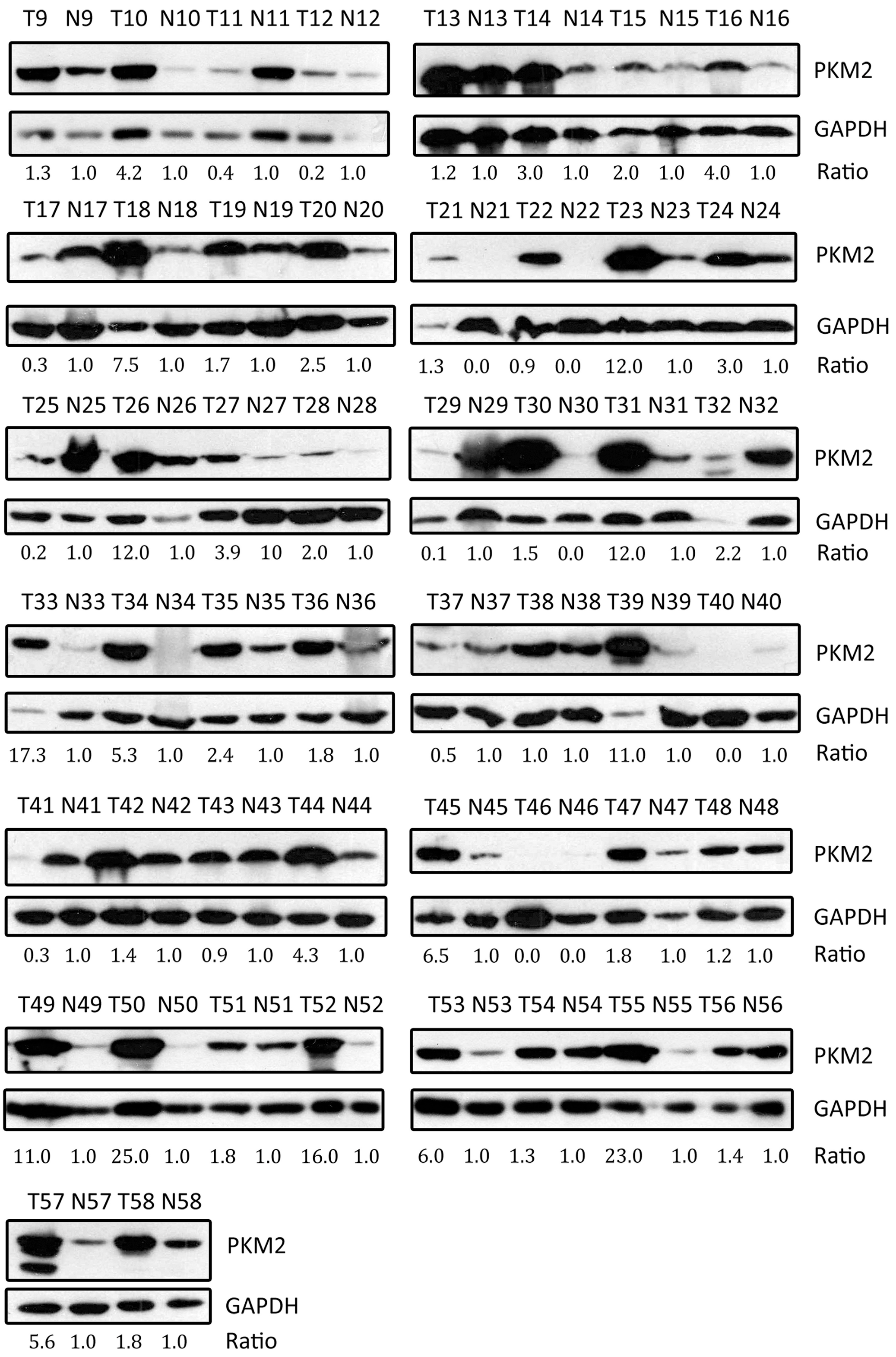

Supplementary Figure 1: PKM2 expression in 50 paired HCC fresh tissues and adjacent nontumourous tissues were determined by western blot.

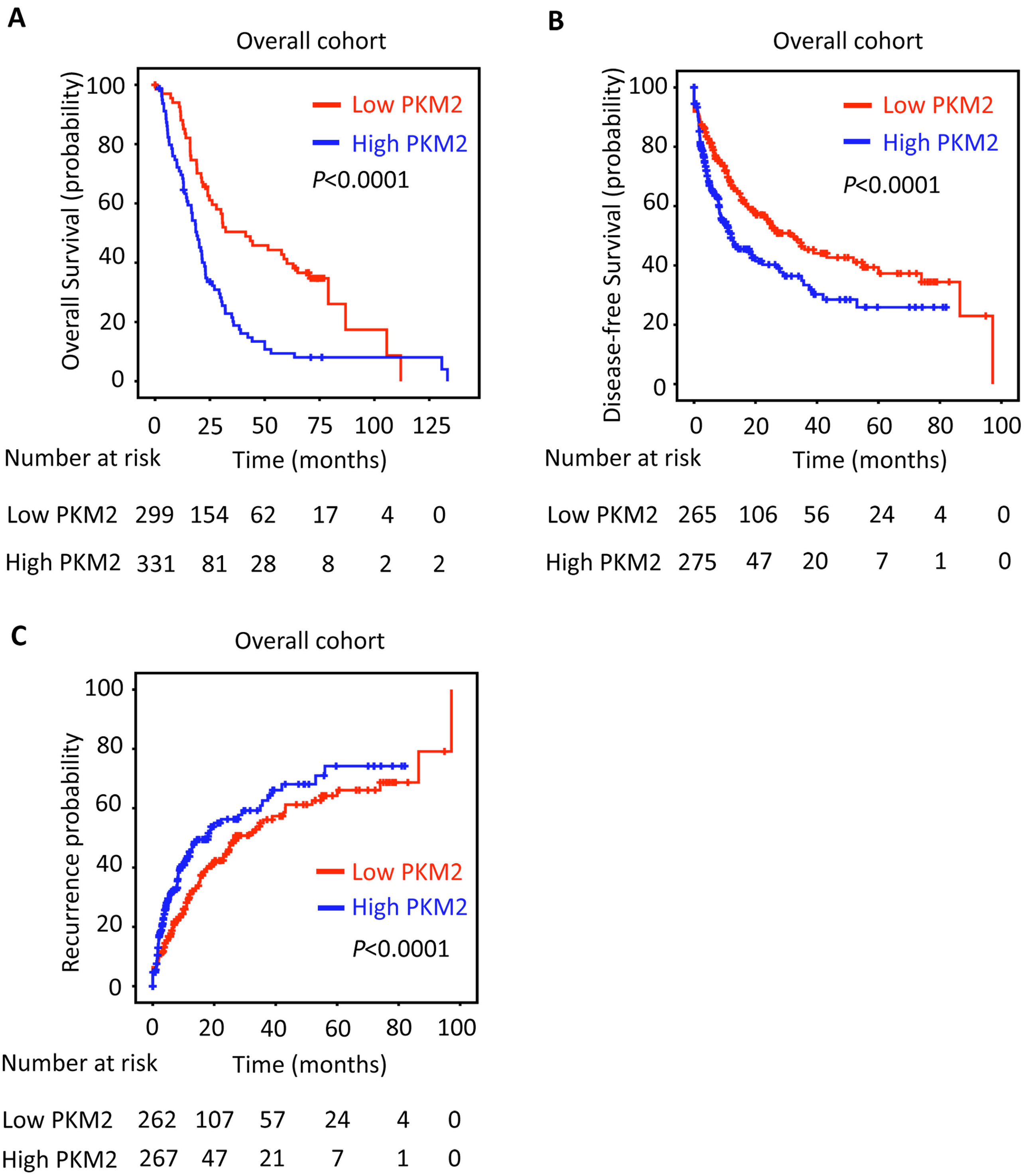

Supplementary Figure 2: Kaplan–Meier's analysis showed the correlation of PKM2 expression and overall survival, disease-free survival and recurrence rate of HCC patients in the overall cohort (*n* = 638, log-rank test).

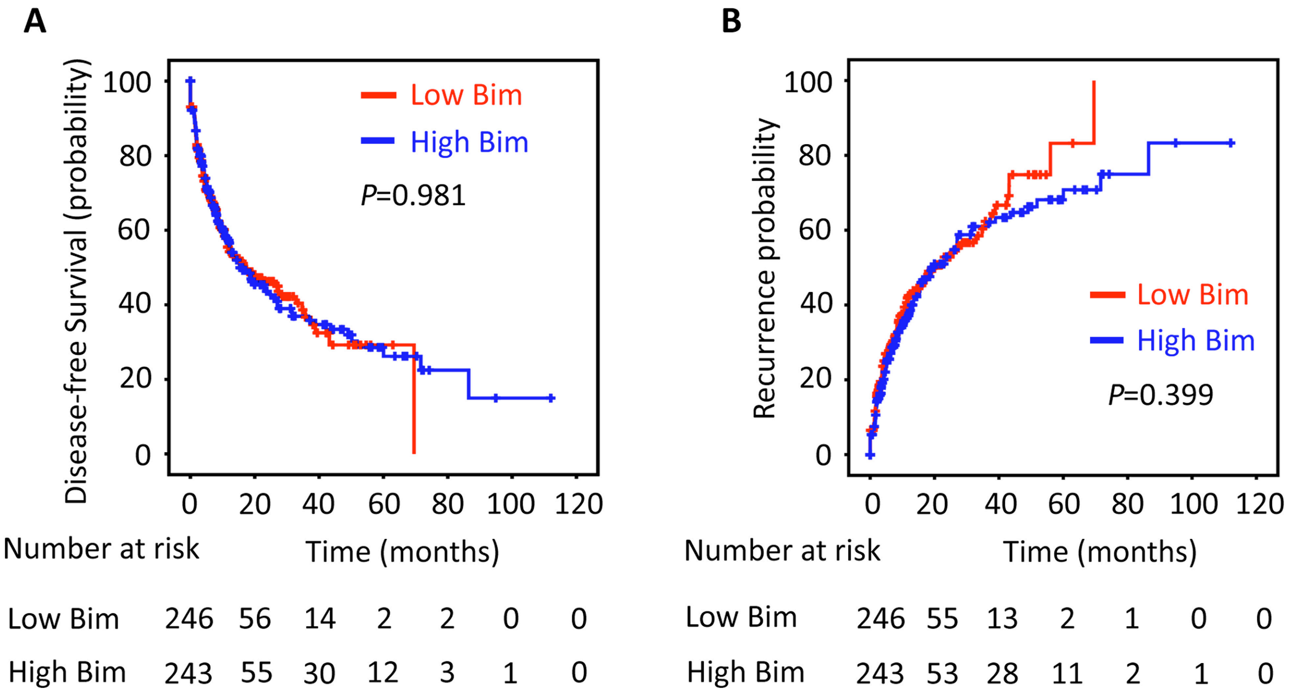

Supplementary Figure 3: Kaplan–Meier analysis of disease-free survival (A) and recurrence rate (B) in 490 cases based on Bim expression was conducted (log-rank test).

Supplementary Table 1: Analysis of clinicopathological parameters in training cohort (*n* = 490) and validation cohort (*n* = 148)

Variable	Training cohort (*n* = 490)	Validation cohort (*n* = 148)	*P* value[a]
Age (years)[b]			0.337
< 49	223 (45.5%)	74 (50.0%)	
≥ 49	267 (54.5%)	74 (50.0%)	
Gender			0.348
Male	441 (90.0%)	137 (92.6%)	
Female	49 (10.0%)	11 (7.4%)	
HBsAg			0.239
Positive	423 (86.3%)	122 (82.4%)	
Negative	67 (13.7%)	26 (17.6%)	
AFP (ng/ml)			0.916
< 20	127 (25.9%)	39 (26.4%)	
≥ 20	363 (74.1%)	109 (73.6%)	
Cirrhosis			0.982
Yes	401 (81.8%)	121 (81.8%)	
No	89 (18.2%)	27 (18.2%)	
Tumor size (cm)			0.734
< 5	126 (25.7%)	36 (24.3%)	
≥ 5	364 (74.3%)	112 (75.7%)	
Tumor multiplicity			**0.049**
Single	293 (59.8%)	75 (50.7%)	
Multiple	197 (40.2%)	73 (49.3%)	
Differentiation			0.718
Well-Moderate	340 (69.4%)	105 (70.9%)	
Poor-undifferentiated	150 (30.6%)	43 (29.1%)	
Stage			0.123
I–II	254 (51.8%)	66 (44.6%)	
III–IV	236 (48.2%)	82 (55.4%)	
Vascular invasion			0.599
Yes	99 (20.2%)	27 (18.2%)	
No	391 (79.8%)	121 (81.7%)	

[a]Chi-square test; [b]Median age; AFP, alpha-fetoprotein; HBsAg, hepatitis B surface antigen.

Supplementary Table 2: Correlation of clinicopathological parameters and PKM2 expression in the training and validation cohort

Variable	Training cohort				Validation cohort			
	All cases	Low expression	High expression	*P* value[a]	All cases	Low expression	High expression	*P* value[a]
Age (years)[b]				**0.037**				1.000
< 49	223	95 (42.6%)	128 (57.4%)		74	34 (50%)	40 (50%)	
≥ 49	267	139 (52.1%)	128 (50%)		74	34 (50%)	40 (50%)	
Gender				0.469				0.055
Male	441	213 (48.3%)	228 (51.7%)		137	66 (48.2%)	71 (47.7%)	
Female	49	21 (42.9%)	28 (57.1%)		11	2 (18.2%)	9 (81.8%)	
HBsAg				0.114				0.373
Positive	423	196(46.3%)	227 (53.7%)		122	54 (44.3%)	68 (55.7%)	
Negative	67	38 (56.7%)	29 (43.3%)		26	14 (53.8%)	12 (46.2%)	
AFP (ng/ml)				**< 0.001**				**0.023**
< 20	127	79 (62.2%)	48 (37.8%)		39	24 (61.5%)	15 (38.5%)	
≥ 20	363	155 (42.7%)	208 (57.3%)		109	44 (40.4%)	65 (59.6%)	
Cirrhosis				0.907				0.548
Yes	401	191 (47.6%)	210 (52.4%)		121	57 (47.1%)	64 (52.9%)	
No	89	43 (48.3%)	46 (51.7%)		27	11 (40.7%)	16 (59.3%)	
Tumor size (cm)				**0.042**				**0.036**
< 5	126	70 (55.6%)	56 (44.4%)		36	22 (61.1%)	14 (38.9%)	
≥ 5	364	164 (45.1%)	200 (54.9%)		112	46 (41.1%)	66 (58.9%)	
Tumor multiplicity				0.094				0.134
Single	293	149 (50.9%)	144 (49.1%)		75	39 (52.0%)	36 (48.0%)	
Multiple	197	85 (43.1%)	112 (56.9%)		73	29 (39.7%)	44 (60.3%)	
Differentiation				0.193				0.317
Well-Moderate	340	169(49.7%)	171 (50.3%)		105	51 (48.5%)	54 (51.4%)	
Poor-undifferentiated	150	65 (43.3%)	85 (56.7%)		43	17 (39.5%)	26 (60.5%)	
Stage				**0.004**				**0.027**
I–II	254	137(53.9%)	117 (46.1%)		66	37 (56.1%)	29 (43.9%)	
III–IV	236	97 (41.1%)	139 (58.9%)		82	31 (37.8%)	51 (62.2%)	
Vascular invasion				**0.001**				**0.002**
Yes	99	32 (32.3%)	67 (67.7%)		27	11 (40.7%)	16 (59.3%)	
No	391	202 (51.7%)	189 (48.3%)		121	57 (47.1%)	64 (52.9%)	

[a]Chi-square test; [b]Median age; AFP, alpha-fetoprotein; HBsAg, hepatitis B surface antigen.

Supplementary Table 3: Univariate and multivariate analysis of clinicopathological and PKM2 for overall survival in the training and validation cohort

Variables	Univariate analysis		Multivariate analysis	
	HR (95% CI)	*P* value	HR (95% CI)	*P* value
**Training cohort (*n* = 490)**				
Age (< 49 vs. ≥ 49 years)	0.883 (0.733–1.064)	0.190		
Gender (female vs. male)	0.970 (0.715–1.317)	0.846		
HBV (positive vs. negative)	1.024 (0.780–1.345)	0.864		
Tumor size (< 5 vs. ≥ 5 cm)	1.665 (1.340–2.070)	**0.000**	1.373 (1.084–1.740)	**0.009**
Tumor multiplicity (singal vs. multiple)	1.188 (0.984–1.435)	0.073		
Liver cirrhosis (yes vs. no)	0.964 (0.756–1.230)	0.769		
AFP (< 20 vs. ≥ 20 ng/mL)	1.843 (1.474–2.304)	**0.000**	1.438 (1.138–1.817)	**0.002**
Vascular invasion (yes vs. no)	2.012 (1.600–2.531)	**0.000**	1.579(1.233–2.023)	**0.000**
Tumor differentiation	1.298 (1.060–1.588)	**0.011**	1.199 (0.977–1.471)	0.082
TNM (I–II vs. III–IV)	1.600 (1.328–1.929)	**0.000**	1.164 (0.941–1.440)	0.161
PKM2 expression (low vs. high)	1.675 (1.389–2.019)	**0.000**	1.442 (1.189–1.750)	**0.000**
**Validation cohort (*n* = 148)**				
Age (< 49 vs. ≥ 49 years)	1.063 (0.741–1.524)	0.742		
Gender (female vs. male)	0.906 (0.441–1.862)	0.789		
HBV (positive vs. negative)	0.947 (0.602–1.489)	0.813		
Tumor size (< 5 vs. ≥ 5 cm)	1.955 (1.244–3.072)	**0.004**	1.792 (1.132–2.838)	**0.013**
Tumor multiplicity(singal vs. multiple)	1.183 (0.824–1.699)	0.361		
Liver cirrhosis (yes vs. no)	1.078 (0.658–1.765)	0.766		
AFP (< 20 vs. ≥ 20 ng/mL)	1.678 (1.083–2.601)	**0.021**	1.420 (0.900–2.241)	0.131
Vascular invasion (yes vs. no)	2.496 (1.597–3.899)	**0.000**	1.939 (1.216–3.092)	**0.005**
Tumor differentiation	0.964 (0.644–1.444)	0.859		
TNM (I–II vs. III–IV)	1.371 (0.953–1.973)	0.089		
PKM2 expression (low vs. high)	2.182 (1.497–3.180)	**0.000**	1.865 (1.263–2.753)	**0.002**

Supplementary Table 4: Univariate and multivariate analysis of clinicopathological and PKM2 for disease-free survival in the training and validation cohort

Variables	Univariate analysis		Multivariate analysis	
	HR (95% CI)	*P* value	HR (95% CI)	*P* value
**Training cohort (*n* = 490)**				
Age (< 49 vs. ≥ 49 years)	0.951 (0.736–1.230)	0.703		
Gender (female vs. male)	1.110 (0.733–1.682)	0.621		
HBV (positive vs. negative)	1.136 (0.770–1.677)	0.520		
Tumor size (< 5 vs. ≥ 5 cm)	1.237 (0.929–1.649)	0.146		
Tumor multiplicity(singal vs. multiple)	0.825 (0.631–1.078)	0.159		
Liver cirrhosis (yes vs. no)	0.869 (0.631–1.196)	0.389		
AFP (< 20 vs. ≥ 20 ng/mL)	1.411 (1.049–1.898)	**0.023**	1.276 (0.942–1.729)	0.116
Vascular invasion (yes vs. no)	1.161 (0.831–1.623)	0.382		
Tumor differentiation	1.180 (0.893–1.559)	0.244		
TNM (I–II vs. III–IV)	0.859 (0.755–0.977)	**0.021**		
PKM2 expression (low vs. high)	1.573 (1.214–2.038)	**0.001**	1.496 (1.147–1.951)	**0.003**
**Validation cohort (*n* = 148)**				
Age (< 49 vs. ≥ 49 years)	1.169 (0.733–1.864)	0.512		
Gender (female vs. male)	1.481 (0.678–3.237)	0.325		
HBV (positive vs. negative)	1.016 (0.544–1.899)	0.960		
Tumor size (< 5 vs. ≥ 5 cm)	1.563 (0.889–2.750)	0.121		
Tumor multiplicity(singal vs. multiple)	1.062 (0.666–1.695)	0.800		
Liver cirrhosis (yes vs. no)	1.961 (0.938–4.103)	0.074		
AFP (< 20 vs. ≥ 20 ng/mL)	1.189 (0.708–1.999)	0.513		
Vascular invasion (yes vs. no)	1.393 (0.728–2.667)	0.317		
Tumor differentiation	1.046 (0.825–1.326)	0.713		
TNM (I–II vs. III–IV)	1.083 (0.876–1.340)	0.462		
PKM2 expression (low vs. high)	2.451 (1.493–4.024)	**< 0.0001**		

Supplementary Table 5: Correlation of clinicopathological parameters and PKM2 expression in overall cohort

Variable	Overall cohort			
	**All cases**	**Low expression**	**High expression**	***P* value[a]**
Age (years)[b]				0.066
< 49	297	129 (43.4%)	168 (56.6%)	
≥ 49	341	173 (50.7%)	175 (51.3%)	
Gender				0.142
Male	578	279 (48.3%)	299 (51.7%)	
Female	60	23 (38.3%)	37 (61.7%)	
HBsAg				0.073
Positive	545	250 (45.9%)	295 (54.1%)	
Negative	93	52 (55.9%)	41 (44.1%)	
AFP (ng/ml)				**0.000**
< 20	166	103 (62.0%)	63 (38.0%)	
≥ 20	472	199 (42.2%)	273 (57.8%)	
Cirrhosis				0.852
Yes	522	248 (47.5%)	274 (52.5%)	
No	116	54 (46.6%)	62 (53.5%)	
Tumor size (cm)				**0.005**
< 5	162	92 (56.8%)	70 (43.2%)	
≥ 5	476	210 (44.1%)	266 (55.9%)	
Tumor multiplicity				**0.027**
Single	368	188 (51.1%)	180 (48.9%)	
Multiple	270	114 (42.2%)	156 (57.8%)	
Differentiation				0.106
Well-Moderate	445	220 (49.4%)	225 (50.6%)	
Poor-undifferentiated	193	82 (42.5%)	111 (57.5%)	
Stage				**0.000**
I–II	320	174 (54.4%)	146 (45.6%)	
III–IV	318	128 (40.3%)	190 (59.7%)	
Vascular invasion				**0.000**
Yes	126	37 (29.4%)	89 (70.6%)	
No	512	265 (51.8%)	247 (48.2%)	

[a]Chi-square test; [b]Median age; AFP, alpha-fetoprotein; HBsAg, hepatitis B surface antigen.

Supplementary Table 6: Univariate and multivariate analysis of clinicopathological and PKM2 for overall and disease-free survival in overall cohort (*n* = 638)

Variables	Univariate analysis		Multivariate analysis	
	HR (95% CI)	*P* value	HR (95% CI)	*P* value
**Overall survival**				
Age (< 49 vs. ≥ 49 years)	0.955 (0.810–1.126)	0.587		
Gender (female vs. male)	0.990 (0.748–1.311)	0.943		
HBV (positive vs. negative)	1.028 (0.815–1.296)	0.818		
Tumor size (< 5 vs. ≥ 5 cm)	1.705 (1.402–2.073)	**0.000**	1.460 (1.186–1.797)	**0.000**
Tumor multiplicity (singal vs. multiple)	1.151 (0.975–1.360)	0.097		
Liver cirrhosis (yes vs. no)	0.992 (0.797–1.233)	0.939		
AFP (< 20 vs. ≥ 20 ng/mL)	1.722 (1.417–2.093)	**0.000**	1.406 (1.149–1.721)	**0.001**
Vascular invasion (yes vs. no)	2.117 (1.726–2.596)	**0.000**	1.722 (1.381–2.146)	**0.000**
Tumor differentiation	1.185 (0.990–1.419)	0.064		
TNM (I–II vs. III–IV)	1.479 (1.253–1.744)	**0.000**	1.084 (0.900–1.304)	0.396
PKM2 expression (low vs. high)	1.749 (1.481–2.066)	**0.000**	1.522 (1.282–1.807)	**0.000**
**Disease-free survival**				
Age (< 49 vs. ≥ 49 years)	1.018 (0.813–1.274)	0.877		
Gender (female vs. male)	1.199 (0.831–1.730)	0.333		
HBV (positive vs. negative)	1.126 (0.811–1.565)	0.478		
Tumor size (< 5 vs. ≥ 5 cm)	1.294 (1.002–1.670)	**0.048**	1.215 (0.940–1.571)	0.137
Tumor multiplicity (singal vs. multiple)	0.873 (0.693–1.099)	0.249		
Liver cirrhosis (yes vs. no)	1.022 (0.763–1.369)	0.884		
AFP (< 20 vs. ≥ 20 ng/mL)	1.343 (1.041–1.733)	**0.024**	1.194 (0.920–1.549)	0.183
Vascular invasion (yes vs. no)	1.212 (0.900–1.632)	0.205		
Tumor differentiation	1.130 (0.884–1.443)	0.330		
TNM (I–II vs. III–IV)	0.826 (0.658–1.037)	0.100		
PKM2 expression (low vs. high)	1.719 (1.367–2.161)	**0.000**	1.639 (1.298–2.070)	**0.000**

Supplementary Table 7: Correlation of clinicopathological parameters and Bim expression (n = 490)

Variable	Overall cohort			
	All cases	Low expression	High expression	*P* value[a]
Age (years)[b]				0.322
< 49	220	105 (47.7%)	115 (52.3%)	
≥ 49	270	141 (52.2%)	129 (47.8%)	
Gender				0.563
Male	442	220 (49.8%)	222 (50.2%)	
Female	48	26 (54.2%)	22 (45.8%)	
HBsAg				0.204
Positive	415	215 (51.8%)	200 (48.2%)	
Negative	75	31 (41.3%)	44 (58.7%)	
AFP (ng/ml)				0.310
< 20	113	52 (46.0%)	61 (54.0%)	
≥ 20	377	194 (51.5%)	183 (48.5%)	
Cirrhosis				0.570
Yes	409	203 (49.4%)	206 (50.6%)	
No	81	43 (53.1%)	38 (46.9%)	
Tumor size (cm)				0.204
< 5	106	59 (55.7%)	47 (44.3%)	
≥ 5	384	187 (48.7%)	197 (51.3%)	
Tumor multiplicity				0.214
Single	310	149 (48.1%)	161 (51.9%)	
Multiple	180	97 (53.9%)	83 (46.1%)	
Differentiation				0.797
Well-Moderate	350	177 (50.6%)	173 (49.4%)	
Poor-undifferentiated	140	69 (49.3%)	71 (50.7%)	
Stage				0.711
I–II	265	131 (49.4%)	134 (50.6%)	
III–IV	225	115 (51.1%)	110 (48.9%)	
Vascular invasion				0.625
Yes	104	50 (48.1%)	54 (51.9%)	
No	386	196 (50.8%)	190 (49.2%)	

[a]Chi-square test; [b]Median age; AFP, alpha-fetoprotein; HBsAg, hepatitis B surface antigen.

Supplementary Table 8: Univariate and multivariate analysis of clinicopathological and Bim for overall and disease-free survival (n = 490)

Variables	Univariate analysis		Multivariate analysis	
	HR (95% CI)	*P* value	HR (95% CI)	*P* value
**Overall survival**				
Age (< 49 vs. ≥ 49 years)	0.868 (0.726–1.039)	0.123		
Gender (female vs. male)	1.044 (0.774–1.408)	0.778		
HBV (positive vs. negative)	1.162 (0.905–1.492)	0.239		
Tumor size (< 5 vs. ≥ 5 cm)	1.386 (1.116–1.721)	**0.003**	1.181 (0.926–1.505)	0.181
Tumor multiplicity (singal vs. multiple)	1.287 (1.069–1.550)	**0.008**	1.026 (0.830–1.267)	0.815
Liver cirrhosis (yes vs. no)	0.682 (0.535–0.868)	**0.002**	0.778 (0.606–0.998)	**0.049**
AFP (< 20 vs. ≥ 20 ng/mL)	1.381 (1.117–1.707)	**0.003**	1.210 (0.973–1.506)	0.087
Vascular invasion (yes vs. no)	1.915 (1.536–2.387)	**0.000**	1.552 (1.217–1.981)	**0.000**
Tumor differentiation	1.298 (1.064–1.584)	**0.010**	1.026 (0.830–1.267)	0.815
TNM (I–II vs. III–IV)	1.693 (1.412–2.030)	**0.000**	1.307 (1.025–1.666)	**0.031**
Bim expression (low vs. high)	0.754 (0.628–0.906)	**0.003**	0.737 (0.612–0.888)	**0.001**
**Disease-free survival**				
Age (< 49 vs. ≥ 49 years)	0.908 (0.707–1.166)	0.449		
Gender (female vs. male)	1.022 (0.670–1.558)	0.921		
HBV (positive vs. negative)	1.026 (0.726–1.450)	0.885		
Tumor size (< 5 vs. ≥ 5 cm)	1.029 (0.769–1.377)	0.846		
Tumor multiplicity(singal vs. multiple)	0.894 (0.684–1.169)	0.413		
Liver cirrhosis (yes vs. no)	0.825 (0.592–1.148)	0.254		
AFP (< 20 vs. ≥ 20 ng/mL)	1.100 (0.824–1.468)	0.518		
Vascular invasion (yes vs. no)	1.182 (0.859–1.627)	0.304		
Tumor differentiation	0.986 (0.740–1.314)	0.924		
TNM (I–II vs. III–IV)	0.861 (0.665–1.114)	0.254		
Bim expression (low vs. high)	0.997 (0.775–1.282)	0.981		
